# On Conicine

**Published:** 1852-06

**Authors:** 


### BUFFALO MEDICAL JOURNAL

AND

### MONTHLY REVIEW.

**VOL. 8.** JUNE, 1852. **NO. 1.**

### ORIGINAL COMMUNICATIONS.

---

ART. I. — *On Conicine.* By M. ORFILA. Translated from the French, by CHAS. A. LEE, M. D.

C[illegible]*cine*, discovered in 1826 by Gieseke, was studied by Geiger in 1831. I[illegible] in every part of the large Hemlock, (*conium maculatum*,) but especially i[illegible] the seeds. It is a colorless, or slightly yellowish liquid, changing [illegible]ction of air, and becoming brown, after a certain time; *its odor*, *hi[illegible]y be compared to that of the urine of mice*, affects the head and exci[illegible] tears; its taste is acid, and its sp. gravity, less than that of water, is [illegible]. *It changes to a deep blue, reddened turnsal paper.*

It is volatile at about 170 degrees (*centigrade.*) Heated in contact with the air, it gives off white vapors, having a strong smell of celery, mixed with the odor of the urine of mice. Mixed in water and agitated, it rises to the top, and does not readily dissolve, although both alcohol and ether dissolve it very easily. It perfectly neutralizes weak acids, and gives salts, generally deliquescent and not crystaline. Pure and concentrated sulphuric acid does not change it when cold; when heated, it acquires, at first, a greenish-brown color, then a blood-red, and afterward black. Hydrochloric acid furnishes white vapors with ammonia, and changes it to a violet, on being heated.

Nitric acid communicates to it a topaz color, which does not immediately change by the action of heat. Tannic acid throws down a white precipitate.

It behaves with many of the reagents, like ammonia; thus the bichloride of mercury, and chloride of zinc cause a white precipitate; the gelatinous oxide of zinc is soluble in an excess of conicine; the chloride of platinum furnishes a yellow precipitate, soluble in water; the acetate of copper gives a blue precipitate, gelatinous, less soluble in an excess of conicine. The acetate and sub-acetate of lead do not precipitate it. It furnishes with the proto-chloride of palladium a chocolate-colored precipitate soluble in an excess of conicine.

Conicine, composed of carbon, hydrogen and nitrogen, may be represented by a compound of one equivalent of ammonia, three hydrogen, one nitrogen, and one of carbonated hydrogen, containing twelve equivalents of hydrogen, and nitrogen of carbon, ($H^{12}$. $C^{8}$.)

Conicine may be obtained by the following process: — Heat in a distilling apparatus, eighteen ounces (500 grammes) of the seed of hemlock, bruised and well mixed with about ℥is. caustic potash, and three pints of water. The distilled liquid, containing conicine, ammonia, etc., is saturated with sulphuric acid, and evaporated to the consistence of a soft extract. It is then agitated in a mixture of alcohol and ether, which dissolves the sulphate of conicine, leaving the sulphate of ammonia, etc. The sulphate of conicine is decomposed by potash; the conicine floats upon the surface; it is then decanted and left to rest for a while on chloride of lime, to take up the water when it is distilled off.

### ACTION OF CONICINE ON THE ANIMAL ECONOMY.

*Experiments.* — 1. I administered to a dog of moderate size, twelve drops of conicine, recectly prepared by the process above described. The animal immediately ran about the laboratory, and did not seem incommoded; at the end of a minute, it manifested slight vertigo and feebleness in the hind legs, although he continued to walk; *three minutes* after swallowing the conicine, he fell upon his right side, as if dying; immediately after, he had some slight convulsive movements in the extremities, without opisthotonos; then, the convulsions having ceased, the animal fell down immovable, and very languid, and died *five* minutes after the poisoning.

On opening the body, the digestive canal, the liver, the spleen, the kidneys, the lungs and the heart, offered no alteration, worthy of notice. The blood was partially coagulated. The tongue was pale over its whole extent; the epithelium was easily detached on the parts, which had been touched by the alkali. The fauces, the nasal fossæ, and the trachea, contained a notable quantity of sanguinolent mucus.

2. I gave to another dog a double quantity of the same conicine. The animal died at the end of *two minutes*, after having exhibited the same symptoms as in the preceding case, with this difference, that the vertigo did not last over half a minute, and the slight convulsive movements, were not immediately manifested after the cessation of the vertigo, and the dog fell upon his *left* side. He had neither vomitings nor stools, and the animal made no cries. On opening the body, the organs and the blood were found in the same state, as in the preceding case.

It is difficult to reconcile these facts with those that have been published by Dr. Christison regarding conicine. According to this author, this alkali is a poison of most extraordinary activity, scarcely less so than hydrocyanic acid. In fact, two drops, applied to a wound or to the eye of a dog, a rabbit, or a cat, caused death in less than ninety seconds; and the same quantity, in the form of the hydrochloric, injected into the femoral vein of a cat, often produced death in three seconds, or a little over. Its activity is much increased when combined with acids, especially the hydrochloric. It does not cause coma, whether administered in a free state, or in the form of a salt. It does not in any manner affect the heart. It possesses a local irritant, and its subsequent effects consist entirely in producing paralysis, which is promptly manifested in the muscular system, and which must always destroy life, by paralyzing the muscles of respiration.

Evidently, the conicine employed by Dr. Christison, was not in the same state of purity and concentration, as that with which I experimented.

I desired to ascertain, whether I could obtain, by a different process, a more active conicine; I therefore digested 18℥ of the seeds of the same hemlock in weak sulphuric acid, and after several hours of contact, I filtered it. The blackish liquor was made alkaline by an excess of potash, when it immediately gave off a strong smell of conicine; I then distilled it in a large retort in a sand-bath; the alkaline liquor in the receiver, strongly alkaline, was placed in contact with chloride of lime for twelve hours, when it was distilled upon the chloride, separating the products, so as to first volatilize the ammonia, lest it might change the conicine.

The alkali obtained by this process, was much more active than the former; in fact, a dose of ten drops killed in two minutes, a very large sized and strong dog. The symptoms exhibited by the animal were the same as I have already described in the former cases.

Every thing goes to justify the belief, that still more active conicine may be obtained by saturating with pure sulphuric acid, the liquid condensed in the receiver, evaporating the liquor to one tenth of its volume, decomposing it by potash, dissolving the conicine which is separated, in ether, decanting, evaporating in the open air, in a current of hydrogen gas, and distilling the conicine obtained, on chloride of lime.

### MEDICO-LEGAL RESEARCHES.

*Experiments.* — I made artificial mixtures of broth, albumen, and wine, or of meat, well hashed, currant jelly, tea and coffee. I added several drops of conicine to each of these mixtures. On the other hand I examined separately, the tongue, the fauces, the stomach and matters it contained, the liver, the spleen, the kidneys, the lungs, and the blood of the dogs I had destroyed with conicine; and *I easily detected this alkali*, both in the artificial mixtures, of which I have spoken, or, the tongue, stomach and its contents, the *spleen, kidneys and lungs.* The liver gave but a trace, and it was impossible to extract from it a single trace of blood, as M. Stas had previously observed with regard to nicotine. I noticed, also, that the lungs furnished a much larger quantity of conicine than the liver. However that may be, it results from these researches, 1st. That conicine is absorbed. 2d. That it may be detected in organs, to which it is carried after absorption. Every thing goes to show, also, that it may be easily detected after prolonged inhumation, as is the case with *nicotine.*

By the following processes, conicine may be extracted from the different organs and alimentary mixtures, whatever they may be; and they are precisely the same as those which I have recommended in searching for nicotine.

### FIRST PROCESS.

Alimentary mixtures or animal tissues, cut into small pieces, are to be placed in eight and a half ounces of distilled water, acidulated with from four to six drops of pure concentrated sulphuric acid, afterward to remain for five or six hours on a filter. The liquid is then evaporated by a very gentle heat until it is reduced to one-sixth of its volume, in order to separate a certain quantity of organic matter. I would remark, that during this evaporation, the liquid undergoes a slight change of color, but does not seem to undergo the least decomposition. When the liquid is cool, it is to be agitated with twice its volume of very concentrated alcohol; this menstruum often throws down an additional quantity of organic matter, which, however, occasions no inconvenience, and requires no interference. It is then again filtered, and evaporated, until the alcohol is entirely volatilized; after leaving the liquor to cool, it is saturated and made alkaline by an excess of soda; at the same time a distinct odor of conicine is perceived. The whole is then to be agitated with sulphuric ether for four or five minutes, in a closed tube, the layer of ether is to be separated by the aid of the finger and a funnel, and the ethereal solution left by itself in a small porcelain capsule. The ether is volatilized, and *leaves the conicine behind.* Nothing further is needed, except to distill it in chloride of lime. It is necessary, in order to extract as large a quantity of conicine as possible, to heat a second time by warm alcohol, (concentrated) the solid matter, resulting from the evaporation of the sulphuric liquor, and the first alcoholic treatment; experience has taught me, in fine, that this solid matter very often contains a small proportion of conicine.

SECOND PROCESS.

Instead of submitting to the action of ether, the liquor saturated by an excess of soda, and obtained in the manner described, it may be distilled over a rapid fire, in a retort, to which a receiver is adapted, which is to be placed in cold water; the conicine, as it comes over, is condensed in the receiver; and nothing more is necessary, except to concentrate it, by evaporation over a gentle fire, or what is better still, distill it on chloride of lime.

